# Multimodal Autoencoder–Based Anomaly Detection Reveals Clinical–Radiologic Heterogeneity in Pulmonary Fibrosis

**DOI:** 10.3390/medsci14010076

**Published:** 2026-02-10

**Authors:** Constantin Ghimuș, Călin Gheorghe Buzea, Alin Horațiu Nedelcu, Vlad Florin Oiegar, Ancuța Lupu, Răzvan Tudor Tepordei, Simona Alice Partene Vicoleanu, Ana Maria Dumitrescu, Manuela Ursaru, Gabriel Statescu, Emil Anton, Vasile Valeriu Lupu, Paraschiva Postolache

**Affiliations:** 1 Grigore T. Popa University of Medicine and Pharmacy, 700115 Iasi, Romania; cosghimus@gmail.com (C.G.); vladoiegar@yahoo.com (V.F.O.); ancuta.ignat1@umfiasi.ro (A.L.); razvan.tepordei@umfiasi.ro (R.T.T.); partene.vicoleanu@umfiasi.ro (S.A.P.V.); ana-maria_dumitrescu@umfiasi.ro (A.M.D.); manuela.ursaru@umfiasi.ro (M.U.); gabriel.statescu@umfiasi.ro (G.S.); emil.anton@yahoo.com (E.A.); vasile.lupu@umfiasi.ro (V.V.L.); par.postolache@umfiasi.ro (P.P.); 2 National Institute of Research and Development for Technical Physics, IFT Iași, 700050 Iasi, Romania; calinb2003@yahoo.com; 3 Clinical Emergency Hospital “Prof. Dr. Nicolae Oblu” Iași, 700309 Iasi, Romania; 4 Radiology Clinic, Recovery Hospital, 700661 Iasi, Romania; 5 Radiology Clinic, “Sf Spiridon” County Clinical Emergency Hospital, 700111 Iasi, Romania

**Keywords:** pulmonary fibrosis, multimodal AI, autoencoder, variational autoencoder, anomaly detection, CT imaging, post-infectious fibrosis, deep learning

## Abstract

**Background:** Pulmonary fibrosis (PF) and post-infectious fibrotic lung disease are characterized by marked heterogeneity in radiologic patterns, physiologic impairment, and clinical presentation. Conventional analytic approaches often fail to capture non-linear and multimodal relationships between structural imaging findings and functional limitation. Integrating imaging-derived representations with clinical and functional data using artificial intelligence (AI) may provide a more comprehensive characterization of disease heterogeneity. **Objectives:** The objective of this study was to develop and evaluate a multimodal AI framework combining imaging-derived embeddings and structured clinical data to identify atypical clinical–radiologic profiles in patients with pulmonary fibrosis using unsupervised anomaly detection. **Methods:** A retrospective cohort of 41 patients with radiologically confirmed pulmonary fibrosis or post-infectious fibrotic lung disease was analyzed. Deep imaging embeddings were extracted from baseline thoracic CT examinations using a pretrained convolutional neural network and integrated with standardized clinical and functional variables. A multimodal variational autoencoder (VAE) was trained in an unsupervised manner to learn the distribution of typical patient profiles. Patient-specific anomaly scores were derived from reconstruction error plus latent regularization (β·KL divergence). Associations between anomaly scores, disease severity, and clinical markers were assessed using Spearman rank correlation. **Results:** Anomaly scores were right-skewed (median 26.91, IQR 22.87–32.11; range 19.75–46.18). Patients above the 85th percentile (anomaly score ≥ 33.85) comprised 7/41 (17.1%) of the cohort and occurred across all clinician-assigned severity categories (mild 3, moderate 1, severe 3). Anomaly scores overlapped substantially across severity groups, with similar medians (mild 26.47, moderate 28.55, severe 28.23). Correlations with conventional severity markers were weak and non-significant, including DLCO (% predicted; ρ = −0.25, *p* = 0.115) and FEV_1_ (% predicted; ρ = −0.22, *p* = 0.165), a pattern consistent with anomaly scores reflecting multimodal deviation rather than severity alone, while acknowledging the exploratory nature of the analysis. Highly anomalous patients frequently exhibited discordant clinical–radiologic profiles, including preserved functional capacity despite marked imaging-derived deviation or disproportionate physiological impairment relative to imaging patterns. **Conclusions:** This proof-of-concept study demonstrates that multimodal VAE-based anomaly detection integrating imaging-derived embeddings with clinical data can quantify clinical–radiologic heterogeneity in pulmonary fibrosis beyond conventional severity stratification. Unsupervised anomaly detection provides a complementary framework for identifying atypical multimodal profiles and supporting individualized phenotyping and hypothesis generation in fibrotic lung disease. Given the modest cohort size, these findings should be interpreted as illustrative and hypothesis-generating rather than generalizable.

## 1. Introduction

Pulmonary fibrosis (PF) comprises a broad and heterogeneous group of interstitial lung diseases (ILDs) characterized by progressive scarring of lung parenchyma, destruction of alveolar architecture, and irreversible decline in gas exchange [1]. The clinical spectrum of fibrotic lung disease ranges from slowly progressive forms to rapidly fatal courses, often independent of apparent radiologic or physiologic severity [2]. Among these, idiopathic pulmonary fibrosis (IPF) remains the most studied subtype; however, fibrosis secondary to autoimmune diseases, environmental exposures, or viral infections, including post-COVID-19 fibrotic sequelae, is increasingly recognized [3,4,5]. Despite significant progress in antifibrotic therapies and imaging-based diagnosis, heterogeneity in disease behavior and response to treatment persists [6].

High-resolution computed tomography (HRCT) and, to a lesser extent, thoracic magnetic resonance imaging (MRI) play a pivotal role in the detection and staging of fibrotic lung disease. HRCT provides structural and textural information that guides diagnostic classification and prognostication [7,8]. Classical radiologic features—reticulation, traction bronchiectasis, honeycombing, and ground-glass opacities—are integrated into severity grading systems that correlate with pulmonary function test (PFT) decline and survival [9,10]. However, the relationship between imaging severity and clinical impairment is frequently discordant: patients with extensive fibrotic changes may maintain near-normal functional capacity, while others with mild imaging alterations experience severe dyspnea, reduced diffusion capacity, or hypoxemia disproportionate to structural damage [11,12]. This clinical–radiologic dissociation underscores the multifactorial nature of PF and the limitations of conventional univariate or linear correlation models.

Recent studies have highlighted that PF should be viewed as a multidimensional and multimodal disorder in which inflammation, vascular remodeling, and microstructural changes interact in complex, non-linear ways [13]. Conventional regression approaches and standard imaging scores cannot fully capture these hidden interactions. Moreover, much of the clinically relevant information remains embedded in unstructured sources—such as radiology reports, diagnostic summaries, and clinical documentation—which are infrequently analyzed quantitatively in routine research settings [14,15]. The integration of complementary data representations therefore represents an important challenge in digital pulmonary medicine.

Artificial intelligence (AI), particularly deep learning, has revolutionized medical image analysis and clinical decision support [16,17]. In pulmonary medicine, AI models have been applied to segment fibrotic areas, quantify lesion burden, and predict survival using imaging biomarkers [18]. Yet, most existing approaches treat each data modality—imaging, clinical variables, or derived descriptors—separately. In contrast, autoencoders (AEs)—unsupervised neural networks trained to reconstruct their input—are effective for representation learning and anomaly detection, by identifying cases that deviate from the learned manifold of “typical” data patterns [19]. In biomedical research, AEs have been used to detect outliers, stratify patient phenotypes, and integrate heterogeneous datasets [20,21].

By integrating clinical, functional, and imaging-derived representations, autoencoder-based models offer an innovative strategy for multimodal phenotyping in fibrotic lung disease. Such frameworks can reveal latent sub-phenotypes, quantify discordance between imaging severity and physiology, and highlight patients with atypical disease trajectories [22]. This perspective aligns with the current movement toward precision medicine in ILD, where individualized profiles rather than categorical diagnoses increasingly guide clinical management [23].

In the aftermath of the COVID-19 pandemic, the clinical spectrum of fibrotic lung injury has broadened substantially [24]. Post-COVID-19 fibrotic sequelae often mimic idiopathic or secondary interstitial fibrosis, with diverse functional outcomes and imaging patterns ranging from ground-glass opacities to reticular fibrotic changes [25,26]. This evolving landscape provides a unique opportunity to apply AI-driven, data-integrative methodologies capable of disentangling overlapping clinical phenotypes.

In this study, we introduce a multimodal AI pipeline that integrates imaging-derived representations from thoracic CT with structured clinical and functional variables, using an autoencoder-based framework for anomaly detection and phenotyping. We analyzed a real-world cohort of patients with pulmonary fibrosis and post-infectious fibrotic lung disease. The primary objectives were to

1. Identify atypical clinical–radiologic profiles using unsupervised anomaly detection;
2. Explore multimodal patterns of disease heterogeneity beyond conventional severity stratification;
3. Assess the potential of autoencoder-based modeling for individualized patient phenotyping.

## 2. Materials and Methods

### 2.1. Study Design and Population

This retrospective observational study included patients with radiologically confirmed pulmonary fibrosis or post-infectious fibrotic lung disease who underwent thoracic computed tomography (CT) imaging as part of routine clinical care. The study population comprised patients evaluated in a real-world clinical setting, reflecting the heterogeneity typically encountered in interstitial lung disease practice. No experimental interventions were performed, and all data were analyzed retrospectively.

Patients were categorized into three clinically defined disease severity groups—mild, moderate, and severe—based on multidisciplinary assessment integrating clinical presentation, pulmonary function testing, and radiologic findings. Severity categorization was performed at the time of baseline evaluation and reflected routine clinical decision-making rather than algorithmic thresholds. Each patient was uniquely assigned to a single severity category, with no overlap between groups.

Disease severity (mild, moderate, severe) was assigned at baseline by a multidisciplinary team including pulmonologists and thoracic radiologists as part of routine clinical care. Severity assessment integrated clinical presentation, pulmonary function testing (including DLCO and FEV_1_% predicted), resting oxygen saturation, exercise capacity (six-minute walk test), symptom burden, oxygen requirement, and a qualitative assessment of the extent and pattern of fibrotic changes on CT. No single variable or numerical threshold was used in isolation; instead, severity categories reflected an overall clinical judgment consistent with standard interstitial lung disease practice.

Histopathologic data were not collected systematically in this retrospective, real-world cohort, as surgical lung biopsy was performed only when clinically indicated and not as part of the present study protocol.

For transparency, Table 1 summarizes the typical clinical, functional, and radiologic features considered during severity assignment in this cohort. Severity labels were assigned prior to and independently of all machine-learning analyses, and investigators performing anomaly detection were blinded to severity categories during model development.

Clinical, demographic, and laboratory data were collected from medical records and stored in a structured spreadsheet. Collected variables included age, sex, pulmonary function indices, exercise capacity measurements, inflammatory markers, symptom scores, and treatment-related indicators. To ensure patient confidentiality and data protection, all identifiers were pseudonymized using a unique alphanumeric identifier (Pacient_ID), which enabled secure linkage between clinical data and imaging-derived features without retaining directly identifiable information.

**Figure 1:**
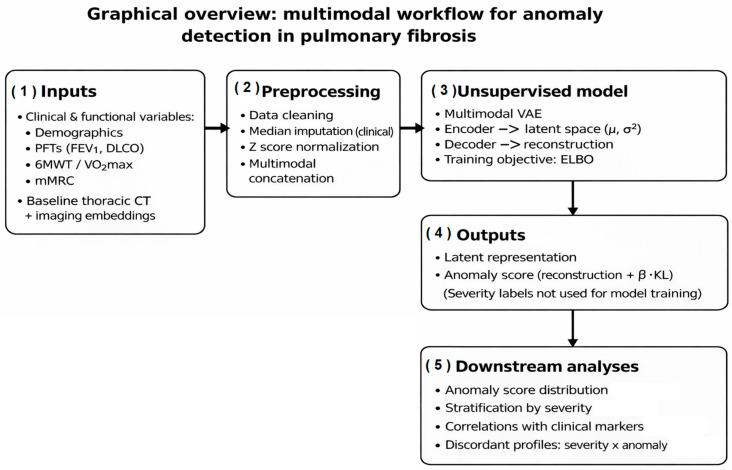
Graphical overview of the multimodal workflow for anomaly detection in pulmonary fibrosis. Clinical and functional variables are combined with imaging-derived deep embeddings extracted from baseline thoracic CT examinations. After data cleaning, normalization, and multimodal feature concatenation, a variational autoencoder (VAE) is trained in an unsupervised manner to learn a probabilistic latent representation of typical patient profiles. The model outputs patient-level latent representations and multimodal anomaly scores derived from reconstruction error and latent regularization. Disease severity labels are not used during model training and are applied only in downstream analyses to interpret anomaly patterns, assess clinical–radiologic discordance, and explore disease heterogeneity.

An overview of the study workflow and the multimodal anomaly detection framework is shown in Figure 1. Clinical and functional variables were integrated with imaging-derived deep representations from baseline thoracic CT examinations and processed through a unified preprocessing pipeline. A multimodal variational autoencoder was then trained in an unsupervised manner to learn latent representations of typical patient profiles and to derive patient-level anomaly scores. Downstream analyses focused on the distribution and clinical interpretation of these anomaly scores in relation to disease severity and functional impairment.

Detailed descriptions of imaging acquisition and clinical data preprocessing are provided in Section 2.2 and Section 2.5, respectively.

### 2.2. Imaging Data Acquisition

Thoracic CT examinations were performed on standard hospital scanners using institution-specific acquisition protocols.

Individual CT examinations frequently contained multiple image series, including non-contrast scans, contrast-enhanced scans, high-resolution reconstructions optimized for lung parenchyma assessment, and ancillary non-diagnostic series such as scout images or protocol sequences. No attempt was made to standardize acquisition retrospectively in order to preserve the ecological validity of the dataset and reflect typical clinical imaging conditions.

All imaging data were available in raw Digital Imaging and Communications in Medicine (DICOM) format and were organized at the patient level prior to preprocessing.

### 2.3. Imaging Preprocessing and Data Reduction

To enable scalable analysis and integration with clinical data, a structured two-stage preprocessing pipeline was applied to the imaging data.

#### 2.3.1. DICOM Handling and Series Selection

Raw DICOM files were retained locally as an archival reference. For analytic purposes, DICOM series were converted to compressed Neuroimaging Informatics Technology Initiative (NIfTI) format (.nii.gz) using *dcm2niix*. This conversion facilitated downstream processing, reduced file redundancy, and ensured compatibility with standard image analysis tools.

Given that individual CT examinations often contained multiple series, a series selection strategy was implemented to identify a single representative diagnostic volume per patient. The selected series was defined as the largest three-dimensional thoracic CT volume based on slice count and in-plane spatial resolution. Single-slice images, scout views, and protocol-related artifacts were explicitly excluded. This strategy ensured consistency across patients while minimizing the inclusion of non-diagnostic or redundant data.

#### 2.3.2. Deep Imaging Feature Extraction

Because voxel-level annotations or segmentation masks of fibrotic lung regions were not available, a weakly supervised feature-based approach was adopted. Rather than using raw volumetric images directly for model training, compact and informative deep imaging representations were extracted and used for downstream analyses.

For each selected CT volume, 32 axial slices were sampled at evenly spaced intervals along the cranio-caudal axis to provide global coverage of the lung parenchyma. Images were resized to 224 × 224 pixels, and lung windowing was applied (window level −600 HU, window width 1500 HU), followed by intensity normalization. Slice intensities were converted to three-channel images to match the input requirements of standard convolutional neural networks. Each slice was then passed through a pretrained ResNet-18 architecture initialized with ImageNet weights. The final fully connected classification layer was removed, and feature vectors were extracted from the final global average pooling layer. This architecture was selected as a lightweight and well-characterized convolutional backbone suitable for feature extraction in small to moderate-sized datasets. Given the limited cohort size and the absence of voxel-level annotations, the network was used as a fixed feature extractor without fine-tuning to reduce the risk of overfitting and to ensure stable representations across patients. Although ImageNet pretraining introduces a domain mismatch between natural images and thoracic CT, prior work has shown that early and intermediate convolutional features can capture generic textural and structural patterns that remain informative in medical imaging applications. In the present proof-of-concept study, we therefore prioritized robustness and model simplicity over maximal domain specialization.

Slice-level embeddings (512 dimensions per slice) were aggregated using average pooling to obtain a single fixed-length imaging feature vector for each patient. This aggregation strategy reduced sensitivity to slice-level noise while preserving global structural and textural information relevant to fibrotic lung disease. The resulting embeddings provided a compact numerical representation of imaging patterns suitable for integration with clinical data.

### 2.4. Multimodal Data Integration

Imaging-derived feature vectors were merged with clinical and demographic data using the pseudonymized Pacient_ID. The resulting multimodal dataset comprised imaging-derived deep embeddings alongside structured clinical and functional variables, as well as clinician-assigned disease severity labels.

Only derived features and tabular data were used for machine-learning analyses. Raw imaging data remained stored locally and were not transferred to cloud-based environments. This approach reduced computational burden and ensured compliance with data governance constraints.

### 2.5. Clinical Data Collection, Cleaning, and Harmonization

Clinical, demographic, and functional data were extracted from electronic medical records and stored in a structured spreadsheet format. A systematic data cleaning and harmonization procedure was applied prior to analysis to ensure internal consistency and analytical robustness.

Column names were translated into English and standardized across severity groups. Variables that appeared under multiple linguistic or orthographic variants were explicitly identified and consolidated into unified columns. Units of measurement were verified for all continuous variables and retained in their original clinical units. Binary and categorical variables were encoded numerically where appropriate to facilitate statistical analysis and modeling.

Administrative fields, such as internal record numbers and raw admission dates, as well as unstructured free-text fields (including narrative diagnostic descriptions), were excluded from modeling to minimize noise, reduce dimensionality, and prevent information leakage. These fields were retained only for auditability and traceability purposes.

### 2.6. Definition of Core and Extended Clinical Feature Sets

Following data cleaning, two complementary clinical feature sets were defined. A core clinical feature set was specified a priori for primary analyses and multimodal modeling, while an extended feature set was reserved for exploratory and sensitivity analyses.

The core feature set was selected based on clinical relevance, interpretability, and data completeness. It included demographic variables (age and sex), measures of gas exchange (resting oxygen saturation, DLCO % predicted, DLCO/VA % predicted), pulmonary function (FEV_1_% predicted), exercise capacity (six-minute walk test distance and maximal oxygen uptake), respiratory muscle strength (maximal inspiratory and expiratory pressures), symptom burden (mMRC dyspnea score), and treatment-related indicators (home oxygen therapy, in-hospital oxygen therapy, inhaled treatment).

Variables exhibiting high proportions of missing values or limited disease specificity were excluded from the core set but preserved in the extended dataset to allow secondary analyses without compromising the robustness of primary results.

### 2.7. Exploratory Data Analysis

Exploratory data analysis was performed to characterize the distributions of clinical variables, assess inter-patient variability, evaluate patterns of missingness, and explore associations with disease severity. EDA was explicitly separated from predictive modeling and was used solely for descriptive and hypothesis-generating purposes.

Continuous variables were summarized using means and standard deviations or medians and interquartile ranges depending on distributional characteristics. Categorical variables were summarized as counts and percentages. All summaries were stratified by disease severity category. Missingness was quantified at both the variable and patient levels, and no imputation was performed prior to EDA to avoid introducing artificial structure into the data.

### 2.8. Statistical Analysis and Confidence Interval Estimation

Given the modest cohort size and the presence of non-normal distributions, non-parametric statistical methods were employed for group comparisons. Differences across severity groups were assessed using Kruskal–Wallis tests for continuous variables and χ^2^ or Fisher’s exact tests for categorical variables, as appropriate.

To complement hypothesis testing and provide clinically interpretable uncertainty estimates, 95% confidence intervals were computed for key continuous variables within each severity group using bootstrap resampling. Effect sizes were reported alongside *p*-values to emphasize the magnitude of observed differences rather than relying solely on statistical significance.

### 2.9. Dimensionality Reduction and Principal Component Analysis

Principal component analysis was applied to the standardized core clinical feature set to explore multivariate structure and relationships among patients. Prior to PCA, all continuous variables were z-score normalized to ensure equal weighting across features.

PCA was used exclusively as an exploratory tool. The first two principal components were retained for visualization, and the proportion of variance explained by each component was reported. Component loadings were examined to identify dominant contributors. PCA was not used for feature selection, classification, or prediction, and no conclusions regarding diagnostic separability were drawn from PCA alone.

### 2.10. Machine Learning Framework

Unsupervised anomaly detection was performed using an autoencoder-based approach to learn the distribution of typical multimodal patient profiles and identify individuals whose clinical–imaging patterns deviated from this learned distribution.

All modeling was performed using Python 3.12.12—based machine-learning libraries in a reproducible computational environment.

### 2.11. Variational Autoencoder (VAE) for Anomaly Detection

**Variational Autoencoder Formulation**

Unlike a standard autoencoder, a Variational Autoencoder (VAE) learns a probabilistic latent representation of the data. Rather than mapping each input deterministically to a single latent vector, the encoder estimates the parameters of a probability distribution in latent space, enabling principled modeling of uncertainty and variability in heterogeneous multimodal data.

Let

$$x_{i}\in R^{d}$$

denote the multimodal feature vector of patient *i*, obtained by concatenating imaging-derived embeddings and clinical variables.

**Encoder**

The encoder approximates the posterior distribution over latent variables as a multivariate Gaussian with diagonal covariance:

$$q_{\theta }\left(z|x\right)=N\left(\mu \left(x\right),diag\left(\sigma^{2}(x)\right)\right)$$

where

- *μ(x)* represents the latent mean;
- *σ^2^(x)* represents the latent variance;
- $z\in R^{k}$, with *k* ≪ *d*, is the latent representation.

The encoder was implemented as a fully connected neural network with nonlinear activations, mapping the input feature space to the parameters $\left(\mu ,\log\sigma^{2}\right)$.

To enable backpropagation through the stochastic sampling process, the reparameterization trick was applied:

$$z\;=\;\mu +\sigma ⊙\epsilon ,\;\;\;\;\;\;\epsilon \sim N\left(0,I\right)$$

where ⊙ denotes element-wise multiplication.

**Decoder**

The decoder reconstructs the input features from the latent variable by modeling the conditional likelihood:

$$p_{\phi }\left(x|z\right)=N\left(\hat{x},I\right),$$

where the reconstructed vector is given by

$$\hat{x}=g_{\phi }(z).$$

The decoder architecture mirrors the encoder in a symmetric fashion, consisting of fully connected layers with ReLU activations that map the latent space back to the original feature dimension.

**Network Architecture**

The VAE employed the following architecture:

**Encoder**

$$x\to Dense(d\to 256)\to ReLU\to Dense\left(256\to 128\right)\to ReLU\to \left(\mu ,\log\sigma^{2}\right)\in R^{k}$$

**Decoder**

$$z\to Dense(k\to 128)\to ReLU\to Dense\left(128\to 256\right)\to ReLU\to \hat{x}\in R^{d}$$

The latent dimensionality was set to *k* = 8 and was intentionally kept low relative to the input feature space to limit model capacity and mitigate overfitting in the context of a modest cohort size.

**Loss Function**

The VAE was trained by minimizing the Evidence Lower Bound (ELBO):

$$L_{VAE}=E_{q_{\theta }\left(z|x\right)}\left[{\left\|x-\hat{x}\right\|}_{2}^{2}\right]+\beta \cdot D_{KL}\left(\left.q_{\theta }\left(z|x\right)\right\|N\left(0,I\right)\right),$$

where the first term corresponds to the reconstruction loss and the second term represents latent space regularization via the Kullback–Leibler (KL) divergence. The KL divergence term is given by

$$D_{KL}=-\frac{1}{2}\sum_{j=1}^{k}\left(1+\log\sigma_{j}^{2}-\mu_{j}^{2}-\sigma_{j}^{2}\right).$$

The hyperparameter *β* controls the trade-off between reconstruction fidelity and latent space regularization. A *β*-weighted formulation was adopted (*β* = 1.0) to encourage smoother and more structured latent representations, prioritizing robust anomaly scoring over maximal reconstruction accuracy.

Because the input feature vector included both continuous and binary variables, reconstruction was implemented using a single mean-squared-error term for simplicity. Future extensions may incorporate mixed likelihoods (e.g., Gaussian for continuous variables and Bernoulli for binary indicators) or feature-specific weighting to further refine reconstruction fidelity.

**Training Procedure**

Prior to modeling, all continuous clinical variables and imaging-derived embeddings were z-score normalized. Binary variables (sex, oxygen therapy, inhaled treatment) were encoded as 0/1 indicators. Multimodal feature vectors were formed by concatenation of clinical and imaging features.

The VAE was trained using the Adam optimizer in pytorch 2.1 (learning rate 1 × 10^−3^, batch size 8) for 200 epochs. Given the modest cohort size, no separate validation set was used. Model training was performed using a fixed random seed to ensure reproducibility.

Anomaly scores were computed on the same cohort used to learn the latent distribution and are interpreted descriptively rather than as generalizable risk estimates. Model stability with respect to random initialization was not systematically assessed and is addressed as a limitation.

**Anomaly Scoring**

For each patient *i*, an anomaly score was defined as

$$A\left(x_{i}\right)={\left\|x_{i}-{\hat{x}}_{i}\right\|}_{2}^{2}+\beta \cdot D_{KL}\left(\left.q_{\theta }\left(z|x_{i}\right)\right\|N\left(0,I\right)\right).$$

Patients with high anomaly scores exhibit multimodal profiles that deviate from the learned distribution of typical disease presentations, indicating atypical clinical–radiologic patterns.

**Rationale for Using a VAE**

The VAE framework was selected because it offers several advantages in the context of heterogeneous clinical–imaging datasets:

- Probabilistic latent space representation;
- Smoother and more structured latent manifolds;
- Improved generalization in small to moderate cohort sizes;
- Interpretable uncertainty through latent variance;
- Principled and quantitative anomaly scoring.

These properties make the VAE particularly suitable for unsupervised identification of atypical disease phenotypes in real-world multimodal medical datasets.

No internal split or external validation cohort was used, and anomaly scores were evaluated only descriptively within the same cohort used for representation learning.

## 3. Results

### 3.1. Clinical Characteristics and Exploratory Data Analysis

A total of 41 patients were included in the final clinical analysis and were stratified into mild, moderate, and severe disease categories. Baseline demographic, clinical, and functional characteristics are summarized in Table 2.

Age distributions were broadly comparable across severity groups, indicating that differences in physiological and functional measures were not driven by age alone. Sex distribution and smoking status showed no marked imbalance between severity categories.

Clear and clinically coherent trends were observed across severity groups for measures of gas exchange, pulmonary function, and exercise capacity. Resting oxygen saturation (SpO_2_) progressively decreased with increasing disease severity. Pulmonary diffusion capacity, expressed as DLCO (% predicted), decreased with increasing disease severity at the group level but showed substantial overlap between moderate and severe categories. This overlap is consistent with the multidimensional nature of clinical severity assignment and known heterogeneity in pulmonary fibrosis, where functional, radiologic, and oxygenation criteria contribute to severity categorization rather than DLCO alone.

Pulmonary function, assessed by FEV_1_ (% predicted), demonstrated a gradual decline across severity categories, although partial overlap between groups was observed.

**Figure 2:**
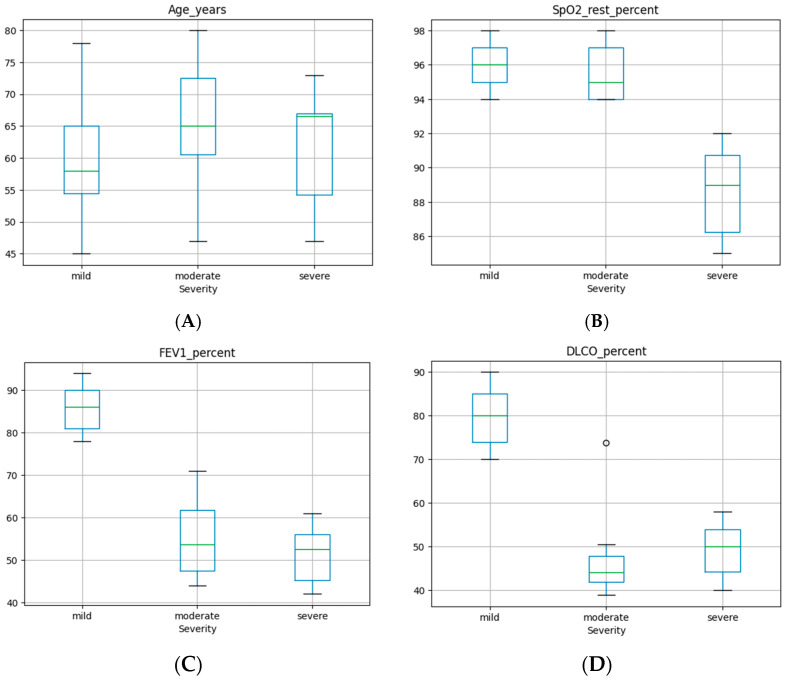

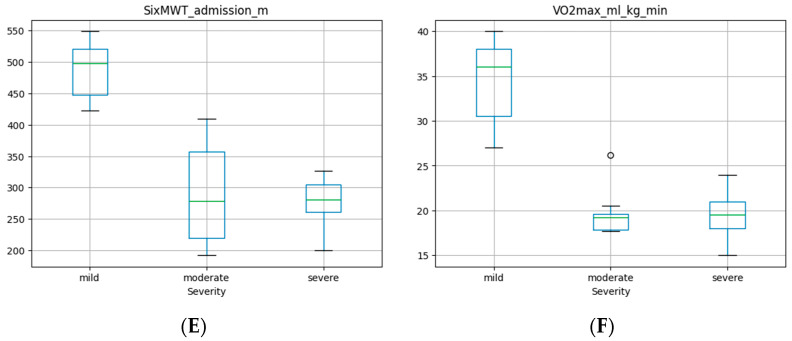
Boxplots of core clinical and functional variables stratified by disease severity (mild, moderate, severe). Panels show distributions of (**A**) age, (**B**) resting oxygen saturation (SpO_2_), (**C**) forced expiratory volume in one second (FEV1, % predicted), (**D**) diffusing capacity of the lung for carbon monoxide (DLCO, % predicted), (**E**) six-minute walk test distance (6MWT, meters), and (**F**) maximal oxygen uptake (VO_2_max, ml·kg^−1^·min^−1^). Boxes represent the interquartile range with median values indicated; whiskers denote the range of observed values. Considerable overlap between severity groups is observed, with a general trend toward lower functional and gas exchange measures in more severe disease categories.

Exploratory boxplot analyses (Figure 2) revealed substantial within-group variability for most clinical parameters, particularly within the moderate severity group. This heterogeneity suggests a broad spectrum of physiological impairment at intermediate disease stages and supports the concept of disease severity as a continuum rather than a set of sharply defined clinical subtypes.

### 3.2. Data Completeness and Missingness Analysis

Data completeness was high across the analyzed clinical variables. All variables included in the core clinical feature set were complete and available for analysis. In the extended dataset, the only variables exhibiting missing values were the COPD Assessment Test (CAT) scores at admission and discharge. These variables were not included in the core feature set and were excluded from all analyses due to substantial missingness. No imputation was performed, given the modest cohort size and the descriptive, hypothesis-generating nature of the study.

### 3.3. Statistical Comparisons Across Severity Groups

Non-parametric statistical testing was performed to assess differences in clinical and functional variables across disease severity groups. Given the small sample size and non-normal distributions, Kruskal–Wallis tests were used for global group comparisons, followed by Dunn’s posthoc tests with Holm correction for multiple comparisons where appropriate. Effect sizes were quantified using Cliff’s delta to provide magnitude-based interpretation of observed differences.

**Global group differences**

Kruskal–Wallis testing revealed statistically significant differences across severity groups for multiple clinically relevant variables, including resting oxygen saturation (SpO_2_), pulmonary diffusion capacity (DLCO and DLCO/VA), pulmonary function (FEV_1_% predicted), exercise capacity (six-minute walk test distance and VO_2_max), symptom burden (mMRC dyspnea score), and respiratory muscle strength (maximal inspiratory pressure).

In contrast, variables related to demographic characteristics did not demonstrate significant global differences, consistent with the descriptive analyses presented in Table 2.

**Pairwise posthoc comparisons**

Posthoc Dunn testing demonstrated that the most pronounced differences were observed between the mild and severe disease groups. Patients with severe disease exhibited significantly lower resting SpO_2_, reduced DLCO, impaired exercise capacity, and higher dyspnea scores compared with patients in the mild group.

Comparisons between mild and moderate disease groups also revealed significant differences for several functional parameters, including DLCO, six-minute walk distance, and VO_2_max, indicating early physiological impairment even at intermediate disease stages.

In contrast, comparisons between moderate and severe groups frequently did not reach statistical significance after correction for multiple testing, despite clear trends toward worsening functional impairment. This finding reflects substantial overlap between these groups and highlights heterogeneity within the moderate severity category.

Treatment-related variables showed expected patterns. The use of supplemental oxygen, both at home and during hospitalization, differed significantly across severity groups, with higher utilization observed in patients with more advanced disease.

**Effect size interpretation**

Effect size analysis using Cliff’s delta supported the clinical relevance of the observed differences. Large effect sizes were observed for comparisons involving exercise capacity (6MWT distance and VO_2_max), gas exchange (DLCO), and resting oxygen saturation when comparing mild and severe disease groups. Moderate effect sizes were observed for comparisons involving mild versus moderate disease, while small-to-moderate effect sizes predominated in comparisons between moderate and severe groups.

Together, these findings indicate that although disease severity is associated with progressive physiological impairment at the group level, substantial inter-individual variability persists, particularly within intermediate disease stages.

Complete statistical test results, including Kruskal–Wallis statistics, Dunn posthoc comparisons, and Cliff’s delta effect sizes, are reported in Supplementary Data File S4.

### 3.4. Principal Component Analysis of Clinical Features

Principal component analysis (PCA) was conducted on the standardized core clinical feature set to explore the multivariate structure of the dataset and to characterize relationships among patients across disease severity categories. Prior to PCA, all continuous clinical variables were z-score normalized to ensure comparable scaling and equal contribution to the analysis. PCA was applied strictly as an exploratory and descriptive method and was not used for classification, prediction, or outcome modeling.

**Figure 3:**
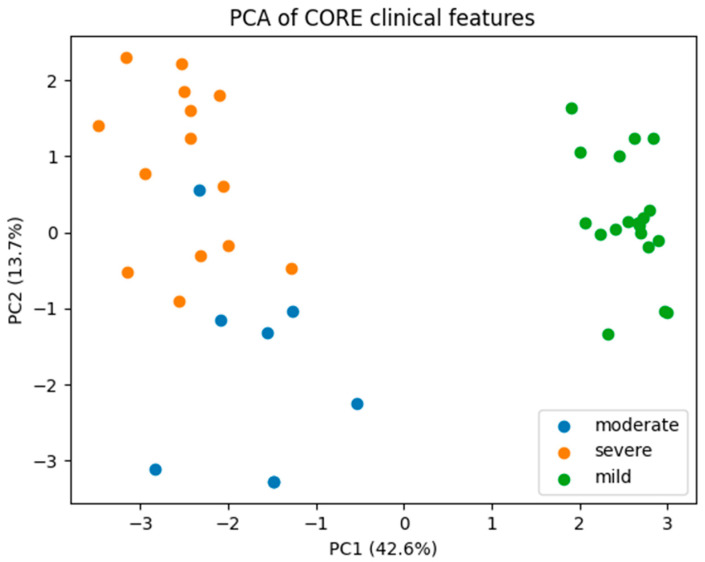
Principal component analysis of core clinical features.Two-dimensional principal component analysis (PCA) of the standardized core clinical dataset. Each point represents an individual patient, colored by disease severity (mild, moderate, severe). The first principal component (PC1) is primarily driven by measures of gas exchange and functional capacity, including DLCO, FEV_1_, SpO_2_, six-minute walk test distance, and VO_2_max. PC2 captures secondary inter-individual variability not directly aligned with disease severity. Partial separation between severity groups is observed along PC1, with substantial overlap, particularly involving moderate cases.

The first principal component explained 67.2% of the total variance, and the second principal component explained an additional 14.2%, such that the first two components together accounted for 81.4% of the variance in the standardized clinical dataset. The distribution of explained variance across principal components is shown in the scree plot (Supplementary Figure S1A).The two-dimensional PCA representation is shown in Figure 3, where each point corresponds to an individual patient projected into the space defined by the first two principal components.Additional diagnostic PCA visualizations, including the correlation structure and joint patient–variable projections, are provided in Supplementary Figure S1B,C.

**Contribution of clinical variables to principal components**

Examination of the PCA loadings revealed that Principal Component 1 (PC1) was predominantly driven by variables reflecting pulmonary gas exchange and exercise capacity. Numerical loadings of individual clinical variables on the first two principal components are reported in Supplementary Table S2. High absolute loadings were observed for six-minute walk test distance, maximal oxygen uptake (VO_2_max), diffusing capacity of the lung for carbon monoxide (DLCO, % predicted), forced expiratory volume in one second (FEV_1_, % predicted), and resting oxygen saturation (SpO_2_). These variables loaded in a consistent direction, indicating that PC1 represents a global axis of physiological and functional impairment.

Lower PC1 scores corresponded to reduced exercise tolerance, impaired gas exchange, and diminished pulmonary function, whereas higher PC1 scores reflected preserved cardiopulmonary performance. The dominance of these variables along PC1 is consistent with the univariate exploratory analyses and non-parametric statistical comparisons, which demonstrated progressive deterioration in these measures with increasing disease severity.

In contrast, Principal Component 2 (PC2) exhibited smaller and more heterogeneous loadings across variables. No single clinical measure dominated this component, suggesting that PC2 captures secondary sources of variability within the cohort. These sources likely reflect inter-individual differences in clinical presentation, baseline characteristics, and physiological profiles that are not directly aligned with disease severity.

**Patient distribution in PCA space**

Projection of patients into the two-dimensional PCA space (Figure 3) revealed a structured but overlapping distribution across severity groups. Patients classified as having severe disease were more frequently located in regions of the PCA space associated with lower PC1 scores, corresponding to more pronounced functional limitation and impaired gas exchange. Conversely, patients with mild disease tended to cluster toward regions characterized by higher PC1 scores, consistent with relatively preserved pulmonary and exercise function.

However, despite these global trends, substantial overlap between severity groups was observed. In particular, the moderate severity group occupied a broad region of the PCA space and overlapped extensively with both mild and severe cases. This overlap was evident along both PC1 and PC2 axes and indicates that patients classified within the same severity category can exhibit markedly different clinical profiles.

The observed overlap suggests that disease severity, as defined by conventional clinical criteria, does not map onto a sharply separable structure in multivariate clinical feature space. Instead, the PCA representation supports the interpretation of disease severity as a continuum, with gradual transitions rather than discrete boundaries between clinical states.

**Relationship to univariate and statistical analyses**

The PCA findings are concordant with the results of univariate exploratory analyses and non-parametric statistical testing. Complete statistical test outputs, including Kruskal–Wallis statistics, posthoc Dunn comparisons, and Cliff’s delta effect sizes, are provided in Supplementary Data File S4. Variables that showed the largest effect sizes and most consistent severity-associated trends in univariate analyses—such as DLCO, 6MWT distance, VO_2_max, and SpO_2_—also contributed most strongly to PC1. At the same time, the extensive overlap observed in PCA space mirrors the partial overlap seen in boxplot distributions and pairwise comparisons, particularly between moderate and severe disease groups.

Thus, PCA provides a complementary multivariate perspective that integrates multiple clinical dimensions simultaneously and highlights both shared patterns and residual heterogeneity within the cohort.

Uncertainty estimates for group-level descriptive statistics are reported as bootstrap-based 95% confidence intervals in Supplementary Table S1.

**Implications for downstream modeling**

The absence of clear separation between severity groups in PCA space (Figure 3) underscores the limitations of relying exclusively on clinical variables for disease stratification. While global trends aligned with increasing disease severity are evident, clinical features alone do not fully capture the complexity and heterogeneity of patient presentations.

These findings provide a strong rationale for the use of more expressive latent representations capable of integrating complementary information sources. In particular, the observed overlap and continuum-like structure in clinical feature space motivated the subsequent application of multimodal latent modeling approaches incorporating imaging-derived representations, with the aim of capturing structural patterns not reflected in standard clinical assessments.

### 3.5. Rationale for Multimodal Latent Modeling

The combined findings from univariate EDA and multivariate PCA underscore the limitations of clinical variables in capturing the full complexity of disease heterogeneity. While severity-associated trends were evident, substantial overlap and inter-patient variability persisted across analyses.

These observations motivated the integration of imaging-derived representations and the application of multimodal latent modeling approaches. By learning joint latent representations from clinical and imaging data, autoencoder-based models offer the potential to capture subtle structural patterns not reflected in conventional clinical measurements. Subsequent analyses therefore focused on multimodal feature integration and unsupervised anomaly detection using variational autoencoder architectures.

### 3.6. Multimodal Variational Autoencoder Training and Convergence

**Figure 4:**
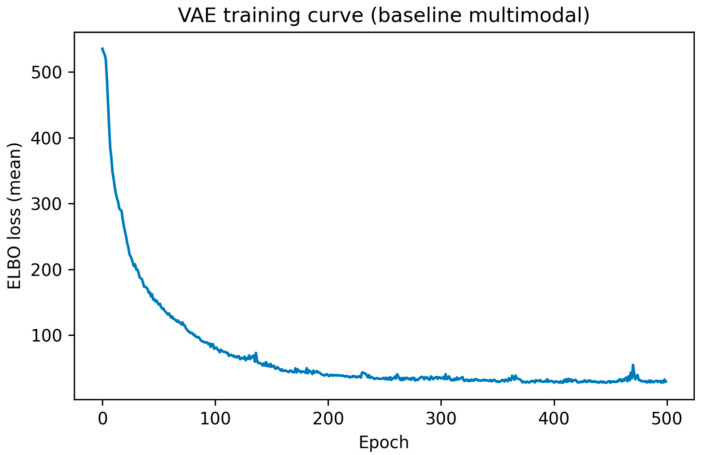
Training curve of the multimodal variational autoencoder, showing mean ELBO loss as a function of training epoch.

The multimodal Variational Autoencoder (VAE) trained on combined clinical variables and imaging-derived embeddings demonstrated stable and well-behaved convergence. As shown in Figure 4, the mean Evidence Lower Bound (ELBO) loss decreased rapidly during the early training epochs, followed by gradual flattening and stabilization in later epochs. This pattern indicates effective learning of a compact latent representation without evidence of numerical instability or divergence.

After approximately 150–200 epochs, further reductions in ELBO were modest, suggesting that the model had reached a stable optimum. Minor fluctuations observed in later epochs likely reflect stochastic variation inherent to mini-batch optimization and latent sampling, rather than systematic overfitting. Overall, the training dynamics support the suitability of the chosen architecture and optimization strategy for modeling multimodal patient data in this cohort.

### 3.7. Distribution of Multimodal Anomaly Scores

**Figure 5:**
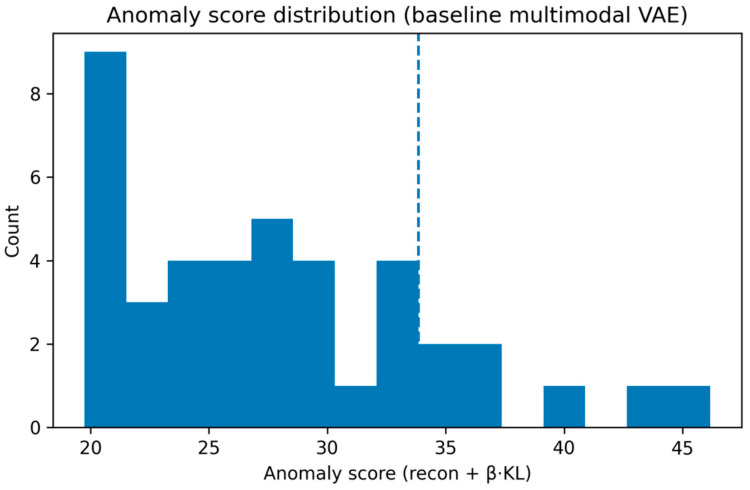
Distribution of multimodal VAE anomaly scores (reconstruction error + β·KL divergence). The dashed vertical line indicates the 85th percentile threshold used to define highly anomalous patients.

Anomaly scores derived from the trained VAE exhibited a right-skewed distribution across the study population (Figure 5). Most patients demonstrated relatively low to intermediate anomaly scores, consistent with profiles well represented by the learned multimodal population manifold. In contrast, a smaller subset of patients showed markedly elevated anomaly scores, reflecting substantial deviation from typical multimodal patterns.

To facilitate descriptive analysis of atypical cases, a percentile-based threshold was applied. Patients exceeding the 85th percentile of the anomaly score distribution—corresponding to approximately 15% of the cohort—were designated as highly anomalous. This threshold was selected a priori as a pragmatic and purely statistical cutoff to highlight extreme multimodal deviations, and it does not represent a clinically defined boundary. Alternative percentile thresholds yielded qualitatively similar patterns, with highly anomalous profiles observed across all disease severity categories, indicating that the main conclusions are not dependent on the specific cutoff selected.

Importantly, anomaly scores were computed in a fully unsupervised manner and did not incorporate disease severity labels or clinical outcomes during model training.

### 3.8. Anomaly Scores Stratified by Disease Severity

**Figure 6:**
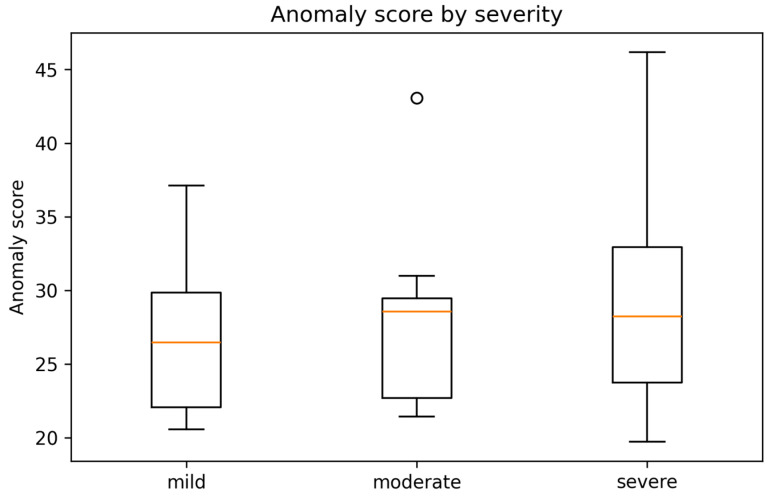
Boxplots of multimodal anomaly scores stratified by disease severity (mild, moderate, severe). Boxes represent interquartile ranges with median values indicated.

When stratified by clinician-assigned disease severity, anomaly scores showed overlapping distributions across mild, moderate, and severe groups (Figure 6). Median anomaly scores were numerically higher in moderate and severe disease categories compared with mild disease; however, substantial within-group variability was observed.

Notably, several patients classified as having mild disease exhibited anomaly scores comparable to, or exceeding, those observed in severe cases. Conversely, some patients with severe disease demonstrated relatively low anomaly scores, indicating multimodal profiles closely aligned with the learned population norm for advanced disease.

These findings indicate that VAE-derived anomaly scores are not simply a surrogate for conventional severity classification. Instead, they capture deviations in multimodal feature space that reflect complex and potentially discordant relationships between imaging-derived representations and clinical or functional measures.

**Figure 7:**
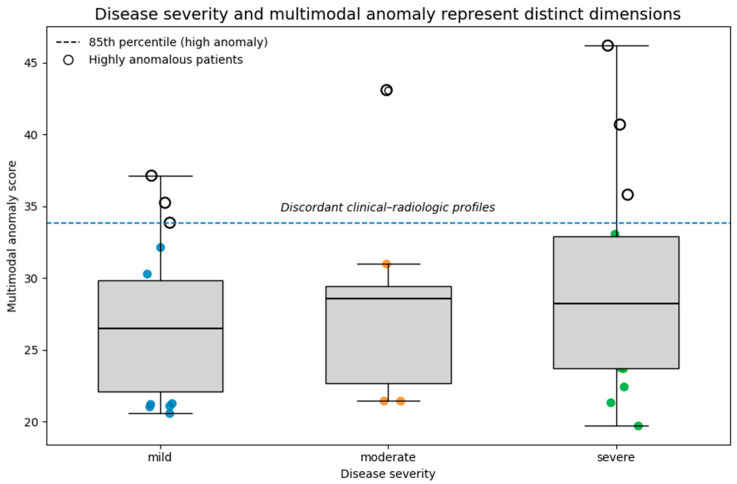
Relationship between disease severity and multimodal anomaly score Boxplots and individual patient points illustrate the distribution of multimodal variational autoencoder-derived anomaly scores across clinician-assigned disease severity categories (mild, moderate, severe). The dashed horizontal line denotes the 85th percentile threshold defining highly anomalous profiles, which are highlighted as hollow circles. While median anomaly scores increase with disease severity, substantial overlap is observed between groups, and highly anomalous patients are present across all severity categories. This pattern highlights discordant clinical–radiologic profiles that are not fully captured by conventional severity stratification.

While Figure 6 summarizes group-level distributions of anomaly scores by disease severity, Figure 7 provides an individual-level and conceptual visualization highlighting highly anomalous patients and illustrating the distinction between severity stratification and multimodal deviation.

To further illustrate the relationship between conventional disease severity and multimodal deviation, a conceptual representation of anomaly scores plotted against severity categories is shown in Figure 7. While median anomaly scores increase across mild, moderate, and severe disease groups, substantial overlap persists, and patients exceeding the 85th percentile anomaly threshold are observed within all severity categories. This representation emphasizes that disease severity and multimodal anomaly capture related but distinct dimensions of disease expression, and that highly discordant clinical–radiologic profiles are not restricted to advanced disease stages.

### 3.9. Association Between Anomaly Scores and Clinical Severity Markers

To further explore the clinical relevance of the anomaly scores, associations with key clinical and functional variables were assessed using Spearman rank correlation analysis. Overall, anomaly scores demonstrated weak-to-moderate correlations with established markers of disease severity.

Spearman correlation coefficients were small in magnitude (|ρ| generally < 0.4), with the strongest negative association observed for DLCO % predicted and the strongest positive association observed for mMRC dyspnea score (Supplementary Table S3). Given the modest cohort size (n = 41), these analyses were interpreted descriptively rather than inferentially.

Negative correlations were observed between anomaly scores and pulmonary diffusion capacity (DLCO % predicted) as well as forced expiratory volume in one second (FEV_1_% predicted), indicating that patients with more impaired pulmonary function tended to exhibit higher multimodal deviation from the population baseline. In contrast, positive correlations were observed between anomaly scores and symptom burden, as measured by the modified Medical Research Council (mMRC) dyspnea score.

Resting oxygen saturation and six-minute walk distance showed minimal or inconsistent associations with anomaly scores, suggesting that the VAE-derived representation captures aspects of disease heterogeneity not fully reflected by individual functional measurements alone.

None of the observed correlations reached conventional thresholds for statistical significance, consistent with the limited sample size and the unsupervised, exploratory nature of the anomaly detection framework. No correction for multiple testing was applied. Nevertheless, the directionality of associations was clinically coherent and aligned with increasing disease burden.

Complete correlation coefficients and *p*-values are reported in Supplementary Table S2.

### 3.10. Characterization of Highly Anomalous Patients

Seven patients (7/41, 17.1%) exceeded the 85th percentile threshold of multimodal anomaly scores and were classified as highly anomalous (Supplementary Table S4). These patients demonstrated heterogeneous and often discordant clinical–radiologic profiles, combining atypical imaging-derived embeddings with either disproportionately preserved or disproportionately impaired clinical and functional measures relative to their assigned disease severity category.

Importantly, highly anomalous patients were not confined to a single severity group. They spanned all severity categories, including three patients classified as mild, one as moderate, and three as severe. This distribution underscores that multimodal deviation is not synonymous with advanced disease and that atypical profiles can emerge across the full spectrum of clinical severity.

Collectively, these findings suggest that multimodal anomaly detection captures dimensions of disease heterogeneity that extend beyond standard clinical stratification, potentially reflecting unique pathophysiological patterns, mixed phenotypes, or early divergent disease trajectories.

For illustration, one highly anomalous patient classified as mild disease (Patient A, Supplementary Table S4) exhibited preserved gas exchange and exercise capacity but marked deviation in imaging-derived embeddings, resulting in a high multimodal anomaly score. In contrast, another highly anomalous patient classified as severe disease (Patient B, Supplementary Table S4) demonstrated pronounced functional impairment with comparatively typical imaging-derived representations. These examples highlight discordant clinical–radiologic profiles that are not fully captured by conventional severity-based categorization.

### 3.11. Summary of Multimodal VAE Findings

Taken together, the multimodal VAE learned a stable latent representation of baseline disease heterogeneity and produced anomaly scores that were only partially aligned with conventional severity classification. The presence of highly anomalous patients across all severity categories underscores the multidimensional nature of fibrotic lung disease and supports the value of integrating clinical and imaging-derived information within a unified latent modeling framework.

These results motivated the subsequent interpretation of anomaly patterns in the context of disease heterogeneity and clinical relevance, as discussed in the following section.

## 4. Discussion

In this study, we applied an unsupervised multimodal representation learning framework to integrate imaging-derived embeddings from thoracic computed tomography with structured clinical and functional data in patients with pulmonary fibrosis and post-infectious fibrotic lung disease. Our results demonstrate substantial clinical–radiologic heterogeneity that is not fully captured by conventional disease severity stratification and show that multimodal anomaly detection provides a complementary perspective on disease expression beyond established severity categories.This distinction is summarized conceptually in Figure 7, which illustrates disease severity and multimodal anomaly as related but non-equivalent dimensions of disease expression at the individual patient level.

This study is not intended to propose a diagnostic or prognostic model, nor to redefine disease severity categories, but rather to explore whether multimodal unsupervised representation learning can reveal clinically familiar yet poorly quantified patterns of disease heterogeneity.

Importantly, given the small cohort size and limited number of highly anomalous cases, observed patterns should be interpreted as illustrative examples of multimodal heterogeneity rather than stable or reproducible phenotypes.

Moreover, disease severity and multimodal anomaly represent related but distinct constructs: severity summarizes the magnitude of impairment, whereas anomaly quantifies deviation from typical multimodal patterns at a given disease stage.

### 4.1. Clinical Heterogeneity and Limitations of Conventional Severity Stratification

Consistent with prior clinical experience, univariate analyses revealed clear group-level trends across disease severity categories for gas exchange, pulmonary function, and exercise capacity. Patients classified as having more severe disease exhibited lower resting oxygen saturation, reduced diffusing capacity, impaired pulmonary function, and diminished exercise tolerance. However, these trends were accompanied by marked within-group variability and substantial overlap between severity categories, particularly between moderate and severe disease.

Multivariate exploration using principal component analysis further emphasized this continuum-like structure. Although the first principal component captured a global axis of physiological impairment driven by gas exchange and functional capacity, patients from different severity groups occupied overlapping regions of clinical feature space. These findings highlight a central challenge in pulmonary fibrosis: severity categories derived from clinical and radiologic assessment summarize population-level trends but incompletely reflect inter-individual variability.

### 4.2. Rationale for Unsupervised Multimodal Modeling

A natural extension of multimodal data integration is supervised prediction, such as attempting to infer imaging severity from clinical variables. However, in fibrotic lung disease, imaging severity is often assigned through multidisciplinary assessment that implicitly incorporates clinical context, functional impairment, and disease trajectory. As a result, imaging labels are not fully independent of clinical inputs, introducing a risk of circularity when used as prediction targets.

Moreover, the substantial overlap observed between severity groups suggests that forcing patients into discrete categories may obscure clinically meaningful heterogeneity. In this setting, optimizing predictive accuracy risks reinforcing existing labels rather than revealing new structure. For these reasons, we adopted an unsupervised approach focused on learning the joint distribution of multimodal patient profiles and identifying deviations from typical patterns.

### 4.3. Multimodal Latent Representations and Anomaly Detection

By integrating imaging-derived embeddings with clinical and functional variables, the multimodal variational autoencoder learned a compact latent representation of baseline disease heterogeneity without supervision or predefined phenotypic labels. The resulting anomaly scores exhibited a right-skewed distribution, with a subset of patients demonstrating marked deviation from the learned population baseline.

Anomaly scores were not confined to patients with the most severe disease. Instead, substantial overlap was observed across mild, moderate, and severe categories, and correlations with conventional severity markers such as DLCO and FEV_1_ were weak. This pattern is consistent with anomaly scores reflecting multidimensional deviation rather than disease severity alone, while also allowing for the possibility that part of the captured variance reflects non–disease-specific factors or measurement noise. An elevated anomaly score should therefore be interpreted as a global measure of multimodal discordance, without implying that deviation is driven predominantly by either imaging or clinical variables alone.

Because imaging inputs were represented by global deep embeddings without regional attribution or correlation with specific CT patterns (e.g., honeycombing or reticulation), the imaging contribution to the anomaly score remains partially opaque and may either dominate or dilute multimodal effects.

### 4.4. Interpretation of Highly Anomalous Patient Profiles

Patients within the top 15% of anomaly scores demonstrated heterogeneous and often discordant clinical–radiologic profiles. Several highly anomalous cases exhibited preserved functional capacity despite marked deviation in imaging-derived representations, while others showed disproportionate physiological impairment relative to their imaging embedding patterns. Such discordance is frequently encountered in clinical practice but is difficult to quantify using conventional metrics.

From an analytic perspective, these cases are often treated as noise or outliers. In contrast, anomaly detection explicitly centers these profiles as objects of interest. Rather than defining new disease subtypes, this approach provides a quantitative framework for identifying patients whose disease expression deviates from expected multimodal patterns. These deviations may reflect differences in compensatory physiology, comorbidity burden, inflammatory activity, or early divergent disease trajectories.As shown in Figure 7, such discordant profiles occur across the full spectrum of clinical severity and are not restricted to advanced disease stages.

Importantly, many of the patients with the highest anomaly scores exhibited clinically recognizable discordance between imaging-derived representations and physiological impairment, as illustrated in Section 3.10, suggesting that a substantial fraction of the anomaly signal corresponds to meaningful clinical heterogeneity rather than purely random variation.

### 4.5. Relation to Prior Work

Previous AI-based studies in pulmonary fibrosis have largely focused on supervised tasks such as fibrosis quantification, outcome prediction, or imaging-based classification. While these approaches have yielded valuable insights, they rely on predefined labels and are often optimized for performance metrics rather than interpretability or heterogeneity characterization [27,28].

In contrast, the unsupervised framework employed in this study does not assume a priori phenotypic categories or outcomes. By modeling the joint distribution of multimodal features, the variational autoencoder emphasizes deviation, discordance, and variability rather than classification accuracy. This perspective aligns with recent guideline and consensus statements highlighting the multidimensional and heterogeneous nature of fibrotic interstitial lung disease, in which clinical course and impairment may not map cleanly onto baseline radiologic severity [29,30,31].

This distinction is particularly relevant in real-world cohorts of modest size and in emerging clinical contexts, such as post-infectious fibrotic lung disease, where standardized labels, long-term outcomes, or large annotated datasets may be unavailable.

### 4.6. Clinical Implications and Future Directions

Although exploratory in nature, our findings suggest several potential clinical applications of multimodal anomaly detection in fibrotic lung disease.

From a clinical perspective, multimodal anomaly scores may be interpreted as

- Indicators of discordance between structural imaging patterns and physiological impairment;
- Markers of atypical or mixed phenotypes rather than disease severity;
- Hypothesis-generating signals for closer follow-up or multidisciplinary review;
- Descriptors of baseline heterogeneity rather than predictors of outcome.

Anomaly scores may help identify patients with discordant clinical–radiologic profiles who warrant closer follow-up, multidisciplinary discussion, or targeted diagnostic evaluation. More broadly, unsupervised latent modeling may support hypothesis generation regarding disease mechanisms, phenotypic variability, and treatment response.

Future work in larger and longitudinal cohorts is needed to evaluate the temporal stability of anomaly scores, their relationship to disease progression and therapeutic response, and their potential role in clinical decision-making. Integration of additional data modalities, such as longitudinal imaging, biomarkers, or patient-reported outcomes, may further enhance the expressiveness and clinical relevance of multimodal latent representations.

For example, a patient classified as having mild disease but exhibiting a high multimodal anomaly score could be flagged for closer longitudinal monitoring or multidisciplinary discussion to assess potential discordance between structural imaging patterns and preserved physiological function. Conversely, a patient with advanced functional impairment but a low anomaly score may represent a more typical disease trajectory within that severity category, providing contextual information rather than prompting intervention. Importantly, this framework is not intended for diagnostic classification, prognostication, or treatment selection and should not be used as a standalone clinical decision-making tool.

### 4.7. Limitations

Several limitations of this study should be acknowledged. First, the cohort size was modest, reflecting the exploratory and real-world nature of the dataset. While this limits statistical power and precludes complex supervised modeling or robust subgroup analysis, it also motivated the use of unsupervised representation learning approaches that do not rely on large labeled datasets. The findings should therefore be interpreted as hypothesis-generating rather than definitive.

In addition, anomaly scores were derived from a variational autoencoder trained on the full cohort and computed on the same dataset and are therefore interpreted descriptively rather than as generalizable risk estimates. Model stability with respect to random initialization was not systematically assessed, and future studies with larger cohorts should evaluate the robustness of anomaly rankings across repeated training runs.

Second, imaging data were acquired retrospectively under routine clinical conditions, resulting in heterogeneity in acquisition parameters, reconstruction kernels, slice thickness, and series composition. Although this variability may introduce noise into imaging-derived representations, it also reflects real-world practice and enhances the external relevance of the proposed framework.

Third, voxel-level annotations or segmentation masks of fibrotic lung regions were not available. Consequently, imaging analysis relied on deep feature embeddings extracted from two-dimensional axial slices rather than region-specific quantitative measurements. While this approach captures global structural and textural information, it does not permit spatial localization of fibrotic patterns or attribution of anomaly scores to specific lung regions. Additionally, imaging-derived features were extracted using a two-dimensional ResNet-18 network pretrained on ImageNet rather than a model pretrained or fine-tuned specifically on thoracic CT data. While this choice helped limit model complexity and reduce the risk of overfitting in a modest-sized cohort without voxel-level labels, the domain mismatch between natural images and lung CT scans may have reduced the specificity of the extracted representations for fibrotic patterns. This limitation is more likely to introduce additional noise into the imaging embeddings than to systematically bias anomaly detection results. Future work in larger cohorts should evaluate feature extractors pretrained or fine-tuned on dedicated thoracic CT datasets and volumetric architectures to improve pathological specificity.

Fourth, only a single baseline CT examination per patient was included in the analysis. Longitudinal imaging and clinical data were not available, precluding assessment of temporal dynamics, disease progression, or treatment response. Future studies incorporating longitudinal data are necessary to evaluate the stability and prognostic relevance of multimodal anomaly scores over time.

Fifth, disease severity categories were assigned based on multidisciplinary clinical and radiologic assessment rather than a single standardized scoring system. While this reflects real-world clinical decision-making, it may introduce subjectivity and limits direct comparability with studies using alternative severity definitions.

Sixth, histopathologic data were not available in a systematic manner. Surgical lung biopsy is performed selectively in routine clinical practice and was not mandated by the study protocol, particularly in patients with advanced disease or significant comorbidities. As a result, we were unable to correlate multimodal anomaly scores with tissue-level features or to use histopathology as an external reference standard for disease heterogeneity in the anomalous cases. While histopathology is often considered a gold standard for characterizing pulmonary fibrosis, its limited availability in real-world cohorts constrains its integration into exploratory multimodal modeling. Future studies combining imaging, clinical data, and histopathology in well-characterized patient subsets may help clarify how anomalydefined profiles relate to underlying fibrotic and inflammatory processes.

Finally, although associations between anomaly scores and selected clinical variables were explored, the study was not designed to establish causal relationships or clinical utility, nor to definitively separate disease-related heterogeneity from non–disease-specific sources of variation. It therefore cannot be excluded that part of the anomaly signal reflects measurement noise, acquisition heterogeneity, or comorbid features not directly related to fibrotic lung disease. External validation in independent cohorts, ideally incorporating longitudinal outcomes and additional biological reference standards, will be required before clinical implementation can be considered.

### 4.8. Strengths

A key strength of this study is the integration of imaging-derived deep representations with structured clinical and functional data within a unified unsupervised modeling framework. By focusing on representation learning and anomaly detection rather than supervised prediction, the analysis avoids circularity inherent in severity-based labels and remains robust to limited sample size. The use of real-world clinical imaging data enhances external relevance, while the separation of exploratory analyses, statistical testing, and latent modeling supports transparency and interpretability. Importantly, the framework highlights clinically familiar yet poorly quantified phenomena—such as discordance between radiologic severity and physiological impairment—providing a principled approach to studying disease heterogeneity in fibrotic lung disease.

## 5. Conclusions

In conclusion, this study demonstrates that unsupervised multimodal modeling integrating imaging-derived embeddings with clinical and functional variables can capture dimensions of disease heterogeneity in pulmonary fibrosis that are not fully reflected by conventional severity stratification. Multimodal anomaly detection identifies patients with atypical clinical–radiologic profiles across all severity categories, emphasizing discordance and individual variability rather than categorical classification. By reframing disease heterogeneity as deviation from a learned multimodal norm rather than misclassification within severity categories, this work highlights the potential of unsupervised multimodal modeling as a complementary lens for understanding fibrotic lung disease.

## Figures and Tables

**Table 1:** Typical clinical, functional, and radiologic features considered during multidisciplinary assignment of disease severity. Values represent common ranges and qualitative patterns observed in routine clinical practice rather than strict numerical cut-offs.

Domain	Mild	Moderate	Severe
Clinical presentation	Minimal dyspnea; symptoms only with exertion	Dyspnea with moderate activity; functional limitation	Dyspnea with minimal activity or at rest
mMRC dyspnea score	0–1	1–2	≥2–3
Resting SpO_2_ (room air)	≥94%	~90–94%	≤90% and/or oxygen-dependent
DLCO (% predicted)	Normal to mildly reduced	Moderately reduced	Moderately to severely reduced
FEV_1_ (% predicted)	Normal or mildly reduced	Mild–moderate reduction	Moderate reduction
6min walk test	Preserved distance (>~400 m)	Reduced distance	Markedly reduced distance
Oxygen therapy	None	May require oxygen with exertion	Frequent or continuous oxygen therapy
CT fibrotic extent	Limited, predominantly basal fibrosis	Bilateral fibrosis of moderate extent	Extensive fibrosis with architectural distortion
CT pattern features	Minimal reticulation, little/no traction bronchiectasis	Reticulation with traction bronchiectasis	Prominent traction bronchiectasis ± honeycombing
Overall MDT impression	Preserved functional reserve	Clear functional impairment	Advanced disease

**Table 2:** Baseline clinical and functional characteristics by disease severity. Values are reported as median [interquartile range] for continuous variables and n (%) for categorical variables.

Variable	Mild	Moderate	Severe
Age, years	58.0 [54.5–65.0]	65.0 [60.5–72.5]	66.5 [54.2–67.0]
SpO_2_ at rest, %	96.0 [95.0–97.0]	95.0 [94.0–97.0]	89.0 [86.2–90.8]
FEV_1_, % predicted	86.0 [81.0–90.0]	53.7 [47.4–61.8]	52.5 [45.2–56.0]
DLCO, % predicted	80.0 [74.0–85.0]	44.0 [42.0–47.9]	50.0 [44.2–54.0]
6MWT distance, m	498.0 [448.0–521.0]	279.0 [220.0–357.0]	280.5 [260.8–304.8]
VO_2_max, ml·kg^−1^·min^−1^	36.0 [30.5–38.0]	19.2 [17.9–19.6]	19.5 [18.0–21.0]
Sex: female, n (%)	9 (47.4%)	3 (37.5%)	6 (42.9%)
Sex: male, n (%)	10 (52.6%)	5 (62.5%)	8 (57.1%)

## Data Availability

The data supporting the findings of this study are not publicly available due to ethical and privacy restrictions related to patient data. The datasets and analysis programs used in this study were stored and processed using Google Colab and Google Drive. Access to the data and computational scripts may be granted upon reasonable request to the corresponding author, Alin Nedelcu (alin.nedelcu@umfiasi.ro).

## Supplementary Materials

The following supporting information can be downloaded at: https://www.mdpi.com/article/10.3390/medsci14010076/s1, Figure S1: *PCA diagnostic plots*, including (A) scree plot of explained variance, (B) correlation circle of clinical variables, and (C) PCA biplot with patient projections colored by disease severity; Table S1: *Bootstrap-based confidence intervals for continuous clinical variables stratified by disease severity*; Table S2: *Loadings of clinical variables on the first two principal components derived from PCA*; Table S3: *Spearman rank correlations between multimodal VAE anomaly scores and selected clinical variables*; Table S4: *Clinical characteristics of patients in the top 15% of multimodal anomaly scores*; Supplementary Data File S1: *Missingness summary for extended clinical variables*; Supplementary Data File S2: *Bootstrap-based confidence interval estimates for continuous clinical variables*; Supplementary Data File S3: *PCA loadings for the first two principal components*; Supplementary Data File S4: *Statistical test outputs including Kruskal–Wallis tests, Dunn post-hoc comparisons, and Cliff’s delta effect sizes*.
