# Supplementary material for: Multimodal Autoencoder–Based Anomaly Detection Reveals Clinical–Radiologic Heterogeneity in Pulmonary Fibrosis
Source: Med Sci (Basel). 2026 Feb 10;14(1):76. doi: 10.3390/medsci14010076 (PMC12921941; doi:10.3390/medsci14010076)
Supplement: Supplementary file 1 [file medsci-14-00076-s001.zip › medsci-4133928-supplementary.pdf]

Supplementary Material

Supplementary Methods

Statistical Analyses

Non-parametric statistical methods were employed throughout exploratory and inferential analyses due to the limited sample size and the non-normal distribution of several clinical variables. Group-wise comparisons across disease severity categories were performed using Kruskal–Wallis tests, followed by Dunn's post-hoc tests with Holm correction where appropriate. Effect sizes were quantified using Cliff's delta to provide magnitude-based interpretation independent of sample size.

Bootstrap-based 95% confidence intervals were estimated using resampling with replacement and were used exclusively for uncertainty quantification of descriptive statistics for continuous clinical variables. No imputation of missing values was performed prior to exploratory analyses, statistical testing, or dimensionality reduction in order to avoid introducing artificial structure into the data.

Prior to principal component analysis (PCA), continuous clinical variables were standardized to zero mean and unit variance.

Handling of Missing Data

All variables included in the core clinical dataset exhibited complete data and were analyzed using a complete-case approach. Within the extended clinical dataset, missing values were observed only for the COPD Assessment Test (CAT) scores at admission and discharge, with approximately 39% missingness for each variable. No imputation was performed, and variables with substantial missingness were excluded from primary exploratory, statistical, and dimensionality reduction analyses.

Supplementary Tables

Supplementary Table S1. Bootstrap-based confidence intervals for continuous clinical variables

Supplementary Table S1 presents non-parametric bootstrap 95% confidence intervals for group-level summary statistics of continuous clinical variables, stratified by disease severity (mild, moderate, severe). Confidence intervals were estimated using resampling with replacement and were computed for both mean and median values. These estimates provide uncertainty quantification for descriptive statistics reported in Table 1.

Only continuous physiological and functional variables are included in this table. Binary and categorical variables (e.g., smoking status, oxygen therapy, inhaled treatment) were intentionally excluded from bootstrap confidence interval estimation.

Variable	Severity	n	Mean	Mean 95% CI	Median	Median 95% CI
Age_years	mild	19	60.6	[56.6–64.7]	58.0	[55.0–65.0]
Age_years	moderate	8	64.9	[56.2–72.5]	65.0	[56.0–80.0]
Age_years	severe	14	62.6	[57.9–67.0]	66.5	[54.0–67.0]
Smoking_status	mild	19	0.4	[0.2–0.6]	0.0	[0.0–1.0]
Smoking_status	moderate	8	0.4	[0.1–0.8]	0.0	[0.0–1.0]
Smoking_status	severe	14	0.4	[0.1–0.7]	0.0	[0.0–1.0]
SpO2_rest_percent	mild	19	95.9	[95.4–96.5]	96.0	[95.0–97.0]
SpO2_rest_percent	moderate	8	95.5	[94.5–96.5]	95.0	[94.0–97.0]
SpO2_rest_percent	severe	14	88.6	[87.4–89.9]	89.0	[86.5–91.0]
mMRC_admission	mild	19	0.6	[0.4–0.8]	1.0	[0.0–1.0]
mMRC_admission	moderate	8	2.4	[2.0–2.8]	2.0	[2.0–3.0]
mMRC_admission	severe	14	2.7	[2.4–3.1]	3.0	[2.0–3.0]
SixMWT_admission_m	mild	19	489.0	[469.7–508.1]	498.0	[453.0–519.0]
SixMWT_admission_m	moderate	8	290.5	[238.4–346.1]	279.0	[220.0–372.0]
SixMWT_admission_m	severe	14	277.6	[257.9–296.2]	280.5	[259.0–304.5]
FEV1_percent	mild	19	86.0	[83.6–88.3]	86.0	[81.0–90.0]
FEV1_percent	moderate	8	55.5	[49.6–62.4]	53.7	[47.4–67.6]
FEV1_percent	severe	14	51.4	[48.1–54.7]	52.5	[45.0–56.0]
DLCO_percent	mild	19	80.0	[77.1–82.8]	80.0	[75.0–85.0]
DLCO_percent	moderate	8	47.8	[42.5–55.9]	44.0	[42.0–50.5]
DLCO_percent	severe	14	49.3	[46.2–52.2]	50.0	[44.0–54.0]
DLCO_VA_percent	mild	19	82.6	[78.9–	84.0	[78.0–

				86.0]		90.0]
DLCO_VA_percent	moderate	8	45.7	[39.7–53.6]	42.6	[38.6–50.7]
DLCO_VA_percent	severe	14	79.2	[75.4–83.1]	78.0	[72.0–86.0]
VO2max_ml_kg_min	mild	19	34.2	[32.3–36.1]	36.0	[31.0–38.0]
VO2max_ml_kg_min	moderate	8	19.7	[18.3–21.7]	19.2	[17.8–20.5]
VO2max_ml_kg_min	severe	14	19.6	[18.1–21.1]	19.5	[18.0–21.0]
MIP_admission_cmH2O	mild	19	90.9	[85.9–95.8]	91.0	[88.0–96.0]
MIP_admission_cmH2O	moderate	8	80.0	[73.6–86.6]	80.5	[68.7–89.8]
MIP_admission_cmH2O	severe	14	94.9	[88.5–100.6]	97.0	[91.0–102.0]
MEP_admission_cmH2O	mild	19	109.3	[103.6–114.9]	110.0	[100.0–120.0]
MEP_admission_cmH2O	moderate	8	110.1	[104.5–116.1]	111.8	[99.2–112.2]
MEP_admission_cmH2O	severe	14	107.1	[100.7–113.6]	108.5	[95.0–116.5]
Home_oxygen	mild	19	0.0	[0.0–0.0]	0.0	[0.0–0.0]
Home_oxygen	moderate	8	0.2	[0.0–0.6]	0.0	[0.0–1.0]
Home_oxygen	severe	14	0.5	[0.2–0.8]	0.5	[0.0–1.0]
Hospital_oxygen	mild	19	0.0	[0.0–0.0]	0.0	[0.0–0.0]
Hospital_oxygen	moderate	8	0.6	[0.2–0.9]	1.0	[0.0–1.0]
Hospital_oxygen	severe	14	1.0	[1.0–1.0]	1.0	[1.0–1.0]
Home_inhaled_therapy	mild	19	0.6	[0.4–0.8]	1.0	[0.0–1.0]
Home_inhaled_therapy	moderate	8	0.9	[0.6–1.0]	1.0	[1.0–1.0]
Home_inhaled_therapy	severe	14	0.5	[0.2–0.8]	0.5	[0.0–1.0]

Supplementary Table S2. Loadings of clinical variables on the first two principal components

Supplementary Table S2 reports the numerical loadings of individual clinical variables on the first two principal components derived from PCA of the standardized core clinical feature set. Positive and negative values indicate the direction and magnitude of each variable's contribution to the respective principal component.

Variable	PC1 loading	PC2 loading
FEV1_percent	0.383	0.026
DLCO_percent	0.377	0.134
VO2max_ml_kg_min	0.371	0.078
SixMWT_admission_m	0.367	0.138
Hospital_oxygen	−0.362	0.114
mMRC_admission	−0.360	0.036
SpO_2_rest_percent	0.288	−0.242
Home_oxygen	−0.238	0.274
DLCO_VA_percent	0.172	0.470
Age_years	−0.066	0.082
MEP_admission_cmH_2O	0.058	−0.238
Smoking_status	−0.032	0.277
Home_inhaled_therapy	0.008	−0.454
MIP_admission_cmH_2O	−0.000	0.491

Variables with higher absolute loadings contribute more strongly to the corresponding principal component. PC1 primarily reflects gas exchange and functional capacity, whereas PC2 captures secondary sources of inter-individual physiological variability.

Supplementary Table S3. Spearman rank correlations between multimodal VAE anomaly scores and clinical variables

This table reports Spearman rank correlation coefficients assessing associations between multimodal VAE-derived anomaly scores and selected clinical and functional variables. No correction for multiple testing was applied due to the exploratory nature of the analysis.

Clinical variable	Spearman ϱ	p-value
DLCO (% predicted)	−0.250	0.115
mMRC dyspnea score	0.239	0.132
FEV_1 (% predicted)	−0.221	0.165
Age (years)	0.134	0.403
VO_2max (ml·kg^{-}◎min^{-}◎)	−0.132	0.411
Six-minute walk distance (m)	0.029	0.858
Resting SpO_2 (%)	−0.002	0.989

Supplementary Table S4. Clinical characteristics of patients in the top 15% of multimodal anomaly scores

This table summarizes clinical and functional characteristics of patients exceeding the 85th percentile of multimodal VAE anomaly scores. Patient identifiers are pseudonymized.

Patient_ID	Severity	Anomaly score	SpO_2 (%)	DLCO (%)	6MWT (m)	VO_2max	mMRC	Age
Forma severa__Maftian V 2023	severe	46.18	92	40.0	305	18.0	4	73
Forma moderata__Lungu S 07.2023	moderate	43.08	94	44.6	319	17.8	2	80
Forma severa__Anghelus D 2023	severe	40.68	92	51.0	327	24.0	4	51
Forma usoara__Moroi E 2022	mild	37.12	96	72.0	498	36.0	1	65
Forma severa__Manolache Maria 2023	severe	35.80	87	41.0	292	21.0	2	54
Forma usoara__Pintilie N 2023	mild	35.24	97	70.0	549	35.0	0	54
Forma usoara__Ciobanu Florin 2022	mild	33.85	97	84	506	40	1	78

Supplementary Figures

Supplementary Figure S1. PCA diagnostic plots

Diagnostic plots supporting the principal component analysis (PCA) of the standardized core clinical feature set. (A) Scree plot showing the proportion of total variance explained by each principal component. (B) Correlation circle illustrating

correlations between original clinical variables and the first two principal components. (C) PCA biplot displaying patient projections in the PC1–PC2 space together with variable loading vectors, with patients colored by disease severity category.

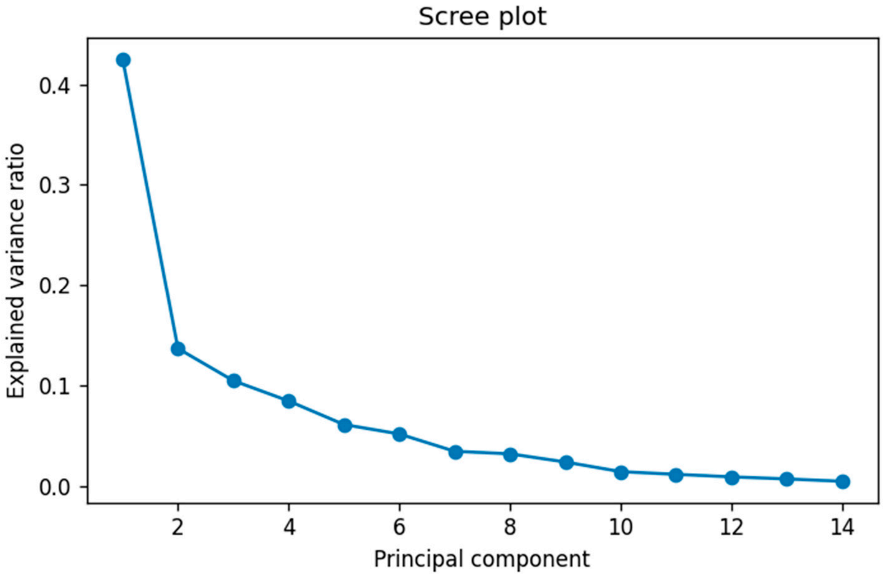

(A)

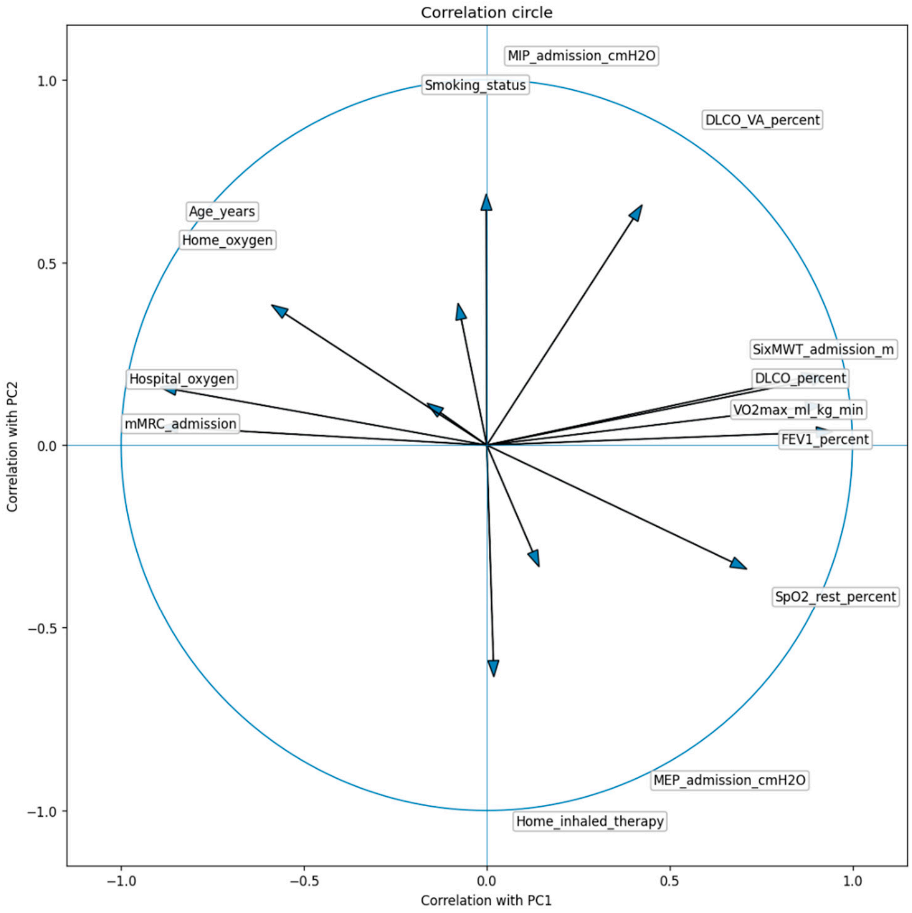
Correlation circle
MIP_admission_cmH2O
Smoking_status
DLCO_VA_percent
Age_years
Home_oxygen
SixMWT_admission_m
Hospital_oxygen
DLCO_percent
VO2max_ml_kg_min
mMRC_admission
FEV1_percent
SpO2_rest_percent
MEP_admission_cmH2O
Home_inhaled_therapy
Correlation with PC2
Correlation with PC1
1.0
0.5
0.0
−0.5
−1.0
−1.0
−0.5
0.0
0.5
1.0

(B)

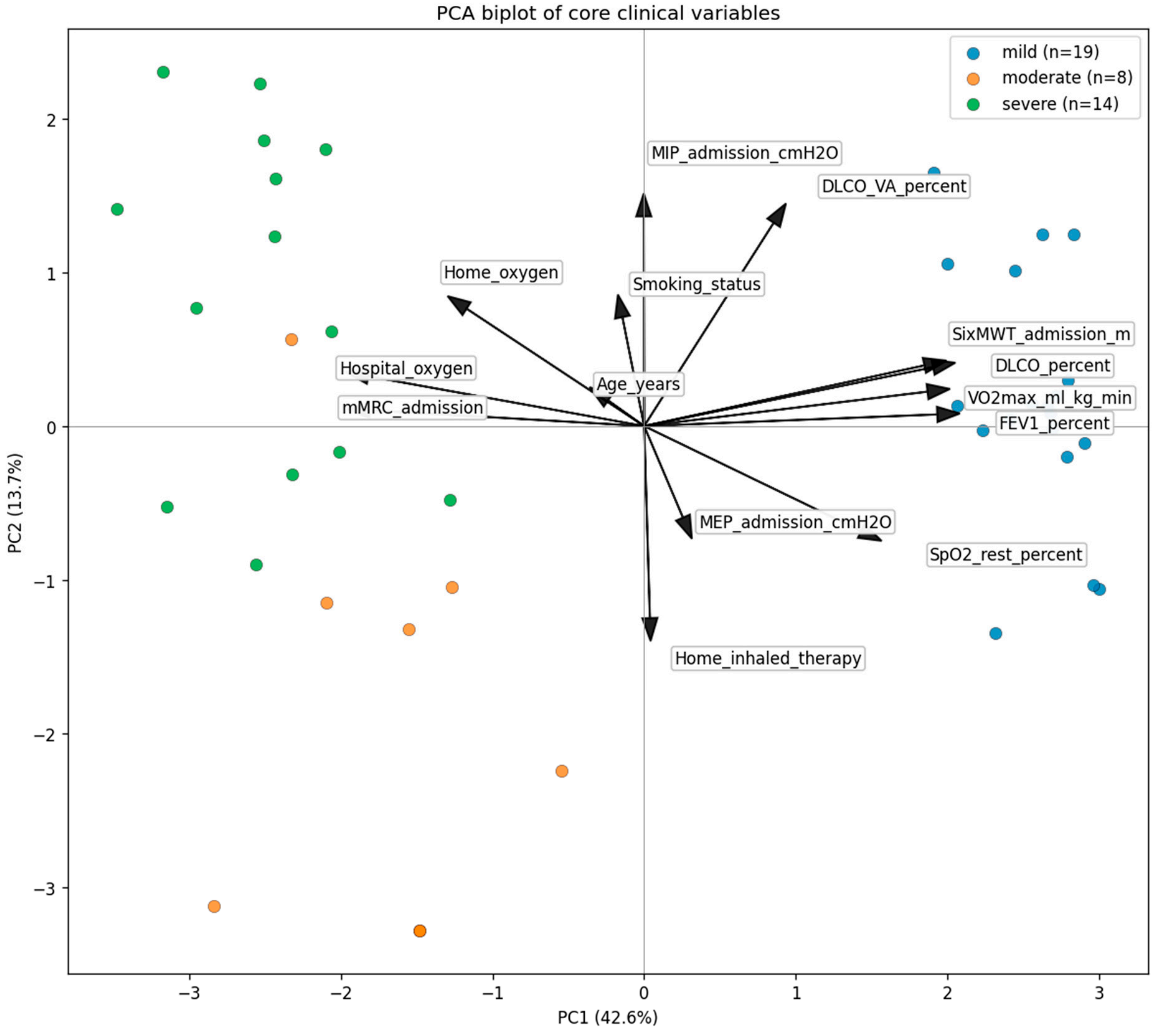

(C)

Supplementary Data Files

Supplementary Data File S1. Missingness summary for extended clinical variables. Supplementary Data File S2. Bootstrap-based confidence interval estimates for continuous clinical variables.

Supplementary Data File S3. PCA loadings for the first two principal components.

Supplementary Data File S4. Statistical test outputs including Kruskal–Wallis tests, Dunn post-hoc comparisons, and Cliff's delta effect sizes.
